# Odds of Stage IV Bone Cancer Diagnosis Based on Socioeconomic and Geographical Factors: A National Cancer Database (NCDB) Review

**DOI:** 10.7759/cureus.34819

**Published:** 2023-02-09

**Authors:** Kevin M McMahon, Vincent Eaton, Kishan K Srikanth, Connor Tupper, Matthew Merwin, Matthew Morris, Peter T Silberstein

**Affiliations:** 1 Medical Education, Creighton University School of Medicine, Omaha, USA; 2 Medicine, Creighton University School of Medicine, Omaha, USA; 3 Internal Medicine, Creighton University School of Medicine, Omaha, USA; 4 Oncology, Creighton University School of Medicine, Omaha, USA

**Keywords:** socioeconomic & geographical factors, ncdb, chordoma, chondrosarcoma, ewing sarcoma, osteosarcoma, cancer staging

## Abstract

Background: There are significant differences in prognosis for osteosarcoma, Ewing sarcoma, chondrosarcoma, and chordomas based on the stage at diagnosis. The five-year survival of these bone cancers varies from 75-87% at an early stage of diagnosis and falls to 27-55% at a late stage of diagnosis.

Patients and methods: This study retrospectively evaluated the odds of stage I vs stage IV cancer at the time of diagnosis in patients with primary malignant bone tumors (osteosarcoma, chondrosarcoma, Ewing sarcoma and chordoma) diagnosed in the National Cancer Database (NCDB) between 2004 and 2018. Cross tabulations with Chi-square analysis were performed to evaluate frequencies of different socioeconomic and geographical characteristics between patients with stage I vs stage IV cancer. Multivariable binary logistic regression was performed to evaluate relationships between socioeconomic and geographical factors and the odds of stage IV cancer at the time of diagnosis. Statistical significance was set at α = 0.05.

Results: 8882 patients with stage I and 3063 with stage IV primary malignant bone tumors were identified. The odds of stage IV bone cancer at diagnosis are increased for patients on Medicaid (odds ratio [OR] = 1.298, 95% confidence interval [CI]: 1.043-1.616) or Medicare (OR = 1.795, 1.411-2.284). Odds of stage IV bone cancer at diagnosis were decreased with female sex (OR = 0.733, 0.671-0.800), private insurance (OR = 0.738, 0.601-0.905), and those treated at community cancer programs (OR = 0.542, 0.369-0.797), comprehensive cancer program (OR = 0.312, 0.215-0.452), or academic/research facilities (OR = 0.370, 0.249-0.550). No significant relationship was identified between the stage at diagnosis and race, ethnicity, Charlson-Deyo score, or education level. Stage IV cancer at diagnosis showed was proportionally lower in chondrosarcomas (17.1%) and chordomas (2.1%) than osteosarcomas (45.0%) and Ewing sarcomas (35.8%).

Conclusion: Odds of stage IV bone cancer at diagnosis are greater with male sex, Medicaid or Medicare insurance status, or treatment at community cancer programs. Providers should have a low suspicion for additional evaluation when treating patients with symptoms of bone cancer and should be aware of these disparities when treating people in these groups. This is to the authors' knowledge the first such study conducted through the NCDB.

## Introduction

According to the Centers for Disease Control and Prevention, at this time there are currently 31.2 million people in the United States under the age of 65 that are uninsured [1]. Additionally, people without insurance are more likely to forgo regular health visits and screening services as they often lack an established location to obtain medical advice and care [2]. Being uninsured or having substandard health insurance has previously been associated with decreased access to care and less frequent evaluation of the nonspecific symptoms for cancers such as primary bone sarcoma and extremity soft-tissue sarcomas. This has been associated with increased odds of metastasis at the time of diagnosis, decreased likelihood of treatment with limb salvage procedures, and reduced disease-specific survival in patients with these tumors [3-4]. Stage of bone tumors at diagnosis has a significant impact on survival. Five-year survival estimates of early vs late-stage tumors for osteosarcoma are 75% vs 27%, Ewing sarcoma 82% vs 39%, chondrosarcoma 78% vs 22%, and chordomas 87% vs 55% [5]. Previous studies on osteosarcoma have also identified that patients with lower socioeconomic status have increased odds of high-grade tumors and overall worse survival [6-7]. Although primary bone cancers comprise less than 1% of all cancers, approximately 3,900 people in the United States are diagnosed with bone cancer annually and 2,100 people die from associated complications annually [8]. These numbers are likely to increase as the population expands.

This study utilized the National Cancer Database (NCDB) to evaluate the odds of receiving a stage I vs stage IV primary bone tumor diagnosis with regard to race, ethnicity, sex, age, comorbidities, insurance status, household income, education, treatment facility location, treatment facility type, distance to the treatment facility, and metropolitan/urban/rural residence. To the authors' knowledge, this is the first study conducted through the NCDB to evaluate the effects of socioeconomic and geographic factors on tumor staging at the time of bone cancer diagnosis.

The abstract for this paper has previously been submitted to the 2022 American Society of Clinical Oncology (ASCO) symposium.

## Materials and methods

A retrospective review of primary bone tumor cases was conducted using the most recent data available through the NCDB [9]. The NCDB was created to improve patient outcomes and now contains approximately 70% of all new patients in the United States diagnosed with cancer. Professional registrars enter individual-level de-identified data from accredited Commission on Cancer facilities (American College of Surgeons-certified hospitals) [10]. The 2018 Participant User File (PUF) for bone and joint tumors was obtained from the NCDB and contained all data used in this study. The 2018 PUF contained data collected on patients diagnosed between 2004 and 2018.

Patients with primary bone tumors were identified using the International Classification of Diseases for Oncology, Third Edition (ICD-O-3) histology codes. Histology codes 9180-9187 and 9192-9195 indicated osteosarcoma; codes 9220-9221, 9230-9231, and 9240-9243 indicated chondrosarcoma; code 9263 indicated Ewing sarcoma; and codes 9370-9372 indicated chordoma. Initially, 24,401 patients with primary osteosarcoma, chondrosarcoma, Ewing sarcoma, or chordomas without concurrent malignancies were identified, however, this number was reduced to 11,945 (8882 with stage I and 3063 with stage IV) when patients with stage II or III tumors or tumors of unknown stage were filtered out. Additionally, it should be noted that facility location and facility type are suppressed for patients aged 0-39, but all these patients were included in all analyses.

All data were analyzed using SPSS statistical software version 28.0 (IBM Corp., Armonk, NY, USA). Descriptive statistics assessed frequencies of race, Spanish/Hispanic ethnicity, sex, age, comorbidities via Charlson-Deyo score, insurance status, median household income (2008-2012), percent no high school completion (2008-2012), treatment facility location, treatment facility type, distance to treatment facility (great circle distance), metropolitan/urban/rural residence, and primary bone tumor histology. Means were also calculated for age and great circle distance. The Charlson-Deyo score is a weighted score derived from the sum of scores of identified comorbid conditions. Conditions such as myocardial infarction, dementia, diabetes, renal disease, or AIDS are scored from 1-6 points. The weighted Charlson-Deyo score ranges from 0-3 with 0 meaning no comorbidities, 1 meaning a single comorbidity, 2 meaning two comorbidities or a single comorbidity with a weight of 2 (such as renal disease), and 3 meaning the patient has significant comorbidities [9]. Age data were represented as five categories (<18, 18-30, 31-50, 51-70, 71+). Race was reported as three categories (white, black, other/unknown) as all racial categories except white and black represented less than 2% of cases. Spanish/Hispanic origins were divided into three classes (non-Spanish/Hispanic, Hispanic, and other). Great circle distance was reported in four categories (0-24 miles, 25-49 miles, 50-99 miles, and 100+ miles). All other factors were broken down by categories present within the NCDB. Median income, percent no high school completion, urban/rural status, and great-circle distance were collected by the NCDB based on the ZIP code in which the patient resided at the time of diagnosis [10].

Cross tabulations and Pearson Chi-square tests were used to compare frequencies of socioeconomic and geographic factors versus tumor stage at diagnosis. These tests were also used to compare frequencies of tumor stage by histology. Multivariable binary logistic regression models were used to determine associations between tumor stage at diagnosis and socioeconomic and geographic factors. Statistical significance was set at α = 0.05.

## Results

This study identified a mean age at diagnosis of 42.55 ± 21.49 years with a median age of 43.0 years and a peak incidence at 16 years. Patient travel distance had a mean of 78.36 miles and a median of 23.10 miles with a range of 0-3,893 miles. Frequencies for race, Spanish/Hispanic ethnicity, sex, age, comorbidities (Charlson-Deyo score), insurance status, median income (2008-2012), percent no high school completion (2008-2012), treatment facility location, treatment facility type, distance to treatment facility (great circle distance), metropolitan/urban/rural residence, and primary bone tumor histology are listed in Table 1.

**Table 1:** Frequencies and Chi-square comparison of patients with stage I and IV cancer at diagnosis

Variable – (n)	Stage I n (%)	Stage IV n (%)	Total n (%)	p-Value
Race (11,945)				0.166
White	7410 (83.4)	2511 (82.0)	9921 (83.1)	
Black	811 (9.1)	310 (10.1)	1121 (9.4)	
Other	661 (7.4)	242 (7.9)	903 (7.6)	
Ethnicity (11,945)				<0.001
Non-Hispanic	7590 (85.5)	2511 (82.0)	10,101 (84.6)	
Hispanic	895 (10.1)	428 (14.0)	1323 (11.1)	
Unknown	397 (4.5)	124 (4.0)	521 (4.4)	
Sex (11,945)				<0.001
Male	4689 (52.8)	1863 (60.8)	6552 (54.9)	
Female	4193 (47.2)	1200 (39.2)	5393 (45.1)	
Age (11,945)				<0.001
<18	919 (10.3)	1015 (33.1)	1934 (16.2)	
18-30	1405 (15.8)	750 (24.5)	2155 (18.0)	
31-50	2679 (30.2)	481 (15.7)	3160 (26.5)	
51-70	2863 (32.2)	535 (17.5)	3398 (28.4)	
71+	1016 (11.4)	282 (9.2)	1298 (10.9)	
Charlson-Deyo Score (11,945)				<0.001
0	7552 (85.0)	2722 (88.9)	10,274 (86.0)	
1	1012 (11.4)	256 (8.4)	1268 (10.6)	
2	213 (2.4)	64 (2.1)	277 (2.3)	
3+	105 (1.2)	21 (0.7)	126 (1.1)	
Primary Payor at Diagnosis (11,945)				<0.001
Not Insured	377 (4.2)	163 (5.3)	540 (4.5)	
Private Insurance/ Managed Care	5322 (59.9)	1621 (52.9)	6943 (58.1)	
Medicaid	975 (11.0)	664 (21.7)	1639 (13.7)	
Medicare	1707 (19.2)	446 (14.6)	2153 (18.0)	
Other Government	191 (2.2)	55 (1.8)	246 (2.1)	
Unknown	310 (3.5)	114 (3.7)	424 (3.5)	
Median Household Income (11,945)				<0.001
		493 (16.1)	1774 (14.9)	
$38,000-$47,999	$38,000-$47,999	681 (22.2)	2480 (20.8)	
$48,000-$62,999	$48,000-$62,999	717 (23.4)	2760 (23.1)	
$63,000+	$63,000+	957 (31.2)	3745 (31.4)	
Unknown	Unknown	215 (7.0)	1186 (9.9)	
Percent No High School Completion (11,945)				<0.001
>21.0%	1411 (15.9)	594 (19.4)	2005 (16.8)	
13.0-20.9%	1955 (22.0)	742 (24.2)	2697 (22.6)	
7.0-12.9%	2512 (28.3)	844 (27.6)	3356 (28.1)	
<7.0%	2040 (23.0)	669 (21.8)	2709 (22.7)	
Unknown	964 (10.9)	214 (7.0)	1178 (9.9)	
Facility type (11,945)				<0.001
Community Cancer Program	86 (1.0)	51 (1.7)	137 (1.1)	
Comprehensive Community Cancer Program	890 (10.0)	267 (8.7)	1157 (9.7)	
Academic/Research Facility	3560 (40.1)	587 (19.2)	4147 (34.7)	
Integrated Network Cancer Program	878 (9.9)	173 (5.6)	1051 (8.8)	
Suppressed	3468 (39.0)	1985 (64.8)	5453 (45.7)	
Facility Location (11,945)				<0.001
Northeast	1142 (12.9)	239 (7.8)	1381 (11.6)	
South	1965 (22.1)	400 (13.1)	2365 (19.8)	
Midwest	1324 (14.9)	239 (7.8)	1563 (13.1)	
West	983 (11.1)	200 (6.5)	1183 (9.9)	
Suppressed	3468 (39.0)	1985 (64.8)	5453 (45.7)	
Great Circle Distance (11,945)				<0.001
0-24 miles	3930 (44.2)	1571 (51.3)	5501 (46.1)	
25-49 miles	1384 (15.6)	504 (16.5)	1888 (15.8)	
50-99 miles	1187 (13.4)	378 (12.3)	1565 (13.1)	
100+ miles	1424 (16.0)	397 (13.0)	1821 (15.2)	
unknown	957 (10.8)	213 (7.0)	1170 (9.8)	
Urban-Rural Residence (11,945)				<0.001
Metro	7040 (79.3)	2465 (80.5)	9505 (79.6)	
Urban	1070 (12.0)	424 (13.8)	1494 (12.5)	
Rural	112 (1.3)	47 (1.5)	159 (1.3)	
Unknown	660 (7.4)	127 (4.1)	787 (6.6)	
Histology (11,945)				<0.001
Osteosarcoma	1763 (19.8)	1378 (45.0)	3141 (26.3)	
Chondrosarcoma	5104 (57.5)	524 (17.1)	5628 (47.1)	
Ewing sarcoma	444 (5.0)	1098 (35.8)	1542 (12.9)	
Chordoma	1571 (17.7)	63 (2.1)	1634 (13.7)	

The results of the cross-tabulations and Pearson Chi-square tests are also listed in Table 1. Approximately 25.6% of patients were reported to have stage IV cancer at the time of diagnosis. Hispanics, males, patients <31 years of age, patients with no known comorbidities, patients that were uninsured or using Medicaid, patients living in areas with a median income <$48,000, patients living in areas with high school completion rates <87%, patients treated at community cancer programs or comprehensive community cancer programs, patients with a travel distance <50 miles, or those with osteosarcoma or Ewing sarcoma diagnosis had increased proportions of advanced-stage disease at the time of diagnosis. Alternatively, females, patients of non-Hispanic or unknown ethnicity, patients >31 years of age, patients with Charlson-Deyo scores >0, those using private insurance or Medicare, patients with greater rates of high school completion, patients treated at academic/research facilities or integrated cancer network programs, those traveling ≥50 miles, and those with chondrosarcoma or chordoma histology had decreased proportions of advanced-stage disease at the time of diagnosis. There were no statistically significant differences regarding race, facility location, or urban/rural residence status and proportions of tumor staging at diagnosis. Frequencies of tumor stage by tumor histology before filtering are listed in Table 2.

**Table 2:** Census of tumor histology compared to staging

Variable	Osteosarcoma n (%)	Chondrosarcoma n (%)	Ewing Sarcoma n (%)	Chordoma n (%)	P-Value
Stage I	1763 (19.7)	5104 (59.5)	444 (10.4)	1571 (60.5)	<0.001
Stage II	3838 (42.9)	1263 (14.7)	1246 (29.1)	90 (3.5)	
Stage III	180 (2.0)	121 (1.4)	107 (2.5)	19 (0.7)	
Stage IV	1379 (15.4)	524 (6.1)	1098 (25.6)	63 (2.4)	
Unknown or N/A	1780 (19.9)	1572 (18.3)	1386 (32.4)	854 (32.9)	
Total	8939 (100)	8584 (100)	4281 (100)	2591 (100)	

Multivariable analysis

The results of the multivariable binary logistic regression are listed in Table 3. The results showed that the odds of stage IV bone cancer overall were lower for females (odds ratio [OR] = 0.733, 95% confidence interval [CI]: 0.671-0.800), older patients (OR = 0.967, 0.962-0.971), those with private insurance (OR = 0.738, 0.601-0.905), those treated at comprehensive community cancer programs (OR = 0.542, 0.369-0.797), academic/research facilities (OR = 0.312, 0.215-0.452), or integrated cancer network programs (OR = 0.370, 0.249-0.550), and patients living 50-99 miles (OR = 0.750, 0.647-0.869) or 100+ miles from their treating facility (OR = 0.720, 0.622-0.835). Conversely, the odds of stage IV bone cancer overall were higher for patients on Medicaid (OR = 1.298,1.043-1.616) or Medicare (OR = 1.795, 1.411-2.284), and those living in urban areas (OR = 1.305, 1.130-1.507).

**Table 3:** Multivariable odds of stage IV bone cancer at diagnosis *Reference: The reference variable is the variable against which all variables are compared to calculate odds. The odds of the event (stage IV) occurring for other variables are calculated relative to the reference variable. **No odds could be calculated due to the lower census of cases for the variable in this stratum

Variable	Chordoma Odds Ratio (95% CI)	P-Value	Ewing Sarcoma Odds Ratio (95% CI)	P-Value	Chondrosarcoma Odds Ratio (95% CI)	P-Value	Osteosarcoma Odds Ratio (95% CI)	P-Value	Overall Odds Ratio (95% CI)	P-Value
Race										
White	*Reference		*Reference		*Reference		*Reference		*Reference	
Black	2.365 (0.920-6.080)	0.074	1.377 (0.691-2.744)	0.363	1.157 (0.806-1.661)	0.429	0.813 (0.743-1.012)	0.056	0.867 (0.743-1.012)	0.070
Other	1.073 (0.431-2.667)	0.880	1.090 (0.700-1.697)	0.703	0.967 (0.633-1.479)	0.878	0.987 (0.756-1.288)	0.924	0.932 (0.789-1.102)	0.410
Ethnicity										
Non-Hispanic	*Reference		*Reference		*Reference		*Reference		*Reference	
Hispanic	0.563 (0.181-1.752)	0.321	0.979 (0.680-1.411)	0.910	0.887 (0.610-1.289)	0.528	1.066 (0.855-1.329)	0.570	0.961 (0.832-1.110)	0.590
Unknown	0.728 (0.162-3.280)	0.679	1.133 (0.613-2.095)	0.691	0.851 (0.530-1.366)	0.504	1.004 (0.692-1.455)	0.985	0.932 (0.748-1.161)	0.529
Sex										
Male	*Reference		*Reference		*Reference		*Reference		*Reference	
Female	1.349 (0.803-2.266)	0.258	1.053 (0.829-1.338)	0.672	0.823 (0.682-0.992)	0.040	0.654 (0.565-0.757)	<0.001	0.733 (0.671-0.800)	<0.001
Age	0.974 (0.946 -1.002)	0.069	1.016 (1.001 -1.031)	0.037	1.020 (1.009 -1.030)	<0.001	0.995 (0.987-1.003)	0.244	0.967 (0.962-0.971)	<0.001
Charlson-Deyo Score										
0	*Reference		*Reference		*Reference		*Reference		*Reference	
1	1.109 (0.525-2.342)	0.787	0.652 (0.395-1.075)	0.094	0.853 (0.647-1.125)	0.260	1.193 (0.907-1.568)	0.206	0.912 (0.781-1.064)	0.241
2	0.546 (0.072-4.154)	0.560	1.411 (0.453-4.397)	0.553	0.742 (0.430-1.279)	0.282	1.563 (0.838-2.915)	0.160	1.146 (0.850-1.546)	0.371
3	0.966 (0.122-7.653)	0.974	0.847 (0.085-8.487)	0.888	0.929 (0.476-1.810)	0.828	0.679 (0.239-1.932)	0.468	0.856 (0.527-1.391)	0.531
Primary Payor at Diagnosis										
Not Insured	**		*Reference		*Reference		*Reference		*Reference	
Private Insurance/ Managed Care	*Reference		0.419 (0.212-0.829)	0.012	0.481 (0.317-0.732)	<0.001	0.907 (0.655-1.256)	0.556	0.738 (0.601-0.905)	0.004
Medicaid	0.841 (0.305-2.318)	0.738	0.757 (0.371-1.541)	0.443	0.799 (0.483-1.320)	0.381	1.449 (1.030-2.038)	0.033	1.298 (1.043-1.616)	0.020
Medicare	1.626 (0.732-3.612)	0.233	0.762 (0.266-2.180)	0.612	0.704 (0.441-1.123)	0.141	1.662 (1.104-2.500)	0.015	1.795 (1.411-2.284)	<0.001
Other Government	**		0.590 (0.214-1.625)	0.307	0.445 (0.177-1.120)	0.085	0.652 (0.358-1.188)	0.162	0.769 (0.532-1.114)	0.165
Unknown	4.376 (1.479 -12.943)	0.008	0.1.67 (0.071-1.390)	<0.001	0.646 (0.314-1.329)	0.235	1.314 (0.838-2.060)	0.234	0.983 (0.727-1.328)	0.909
Median Household Income										
*Reference		*Reference		*Reference		*Reference		*Reference	
$38,000-$47,999	2.484 (0.824-7.484)	0.106	1.104 (0.737-1.654)	0.631	1.186 (0.848-1.657)	0.319	1.016 (0.795-1.300)	0.898	1.085 (0.931-1.264)	0.297
$48,000-$62,999	1.305 (0.404 -4.220)	0.656	1.252 (0.815 -1.924)	0.305	1.435 (1.008 -2.043)	0.045	0.903 (0.689-1.183)	0.459	1.050 (0.890-1.238)	0.564
$63,000+	1.044 (0.304 -3.586)	0.946	1.553 (0.959 -2.513)	0.073	1.340 (0.894 -2.010)	0.156	1.101 (0.807-1.502)	0.544	1.094 (0.909-1.317)	0.342
Unknown	**		**		**		0.689 (0.058-8.164)	0.768	0.512 (0.061-4.304)	0.538
Percent No High School Completion										
>21.0%	*Reference		*Reference		*Reference		*Reference		*Reference	
13.0-20.9%	0.500 (0.165-1.516)	0.221	0.875 (0.594-1.290)	0.501	1.025 (0.750-1.403)	0.875	0.984 (0.780-1.240)	0.890	0.997 (0.862-1.153)	0.969
7.0-12.9%	1.423 (0.531 -3.817)	0.483	1.114 (0.726 -1.707)	0.622	0.753 (0.533 -1.063)	0.107	0.948 (0.727-1.235)	0.691	0.954 (0.813-1.121)	0.569
<7.0%	1.800 (0.574-5.643)	0.313	0.789 (0.486-1.279)	0.336	0.744 (0.499-1.110)	0.148	0.976 (0.712-1.340)	0.882	0.975 (0.808-1.177)	0.793
Unknown	**		**		**		5.045 (0.146-173.964)	0.370	1.637 (0.117-22.840)	0.714
Facility type										
Community Cancer Program	*Reference		*Reference		*Reference		*Reference		*Reference	
Comprehensive Community Cancer Program	0.557 (0.061-5.114)	0.605	0.800 (0.082-7.761)	0.847	0.819 (0.458-1.464)	0.501	0.901 (0.425-1.910)	0.786	0.542 (0.369-0.797)	0.002
Academic/Research Facility	0.463 (0.053-4.073)	0.487	0.410 (0.047-3.613)	0.422	0.483 (0.273-0.854)	0.012	0.571 (0.280-1.161)	0.122	0.312 (0.215-0.452)	<0.001
Integrated Network Cancer Program	0.325 (0.032-3.299)	0.342	0.273 (0.028-2.688)	0.266	0.562 (0.309-1.024)	0.060	0.799 (0.373-1.710)	0.563	0.370 (0.249-0.550)	<0.001
Facility Location										
Northeast	*Reference		*Reference		*Reference		*Reference		*Reference	
South	0.660 (0.295-1.479)	0.313	0.764 (0.281-2.078)	0.598	0.970 (0.726-1.295)	0.835	0.669 (0.466-0.962)	0.030	0.956 (0.794-1.150)	0.633
Midwest	0.609 (0.257-1.441)	0.259	1.142 (0.381-3.421)	0.812	0.956 (0.698-1.310)	0.780	0.753 (0.499-1.136)	0.176	0.868 (0.707-1.065)	0.176
West	0.842 (0.360-1.972)	0.692	1.189 (0.294-4.803)	0.808	1.146 (0.827-1.589)	0.412	0.672 (0.436-1.037)	0.073	0.915 (0.737-1.136)	0.419
Great Circle Distance										
0-24 miles	*Reference		*Reference		*Reference		*Reference		*Reference	
25-49 miles	0.900 (0.402-2.013)	0.797	1.062 (0.771-1.463)	0.711	0.769 (0.582-1.016)	0.064	0.926 (0.746-1.148)	0.483	0.893 (0.787-1.014)	0.082
50-99 miles	1.067 (0.457-2.494)	0.880	0.974 (0.656-1.447)	0.898	0.667 (0.484-0.919)	0.013	0.973 (0.764-1.240)	0.825	0.750 (0.647-0.869)	<0.001
100+ miles	0.719 (0.322-1.607)	0.422	1.058 (0.714-1.567)	0.778	0.601 (0.423-0.855)	0.005	1.040 (0.813-1.330)	0.755	0.720 (0.622-0.835)	<0.001
unknown	**		**		1.815 (0.030-108.661)	0.775	0.166 (0.013-2.139)	0.169	0.683 (0.143-3.261)	0.633
Urban-Rural Residence										
Metro	*Reference		*Reference		*Reference		*Reference		*Reference	
Urban	1.497 (0.665-3.372)	0.330	1.219 (0.836-1.776)	0.303	1.201 (0.884-1.632)	0.241	1.241 (0.966-1.593)	0.091	1.305 (1.130-1.507)	<0.001
Rural	**		1.224 (0.457-3.280)	0.688	1.330 (0.608-2.911)	0.475	1.110 (0.596-2.064)	0.743	1.360 (0.938-0.938)	0.105
Unknown	0.390 (0.108-1.416)	0.152	1.032 (0.615-1.731)	0.906	0.927 (0.599-1.435)	0.734	0.588 (0.394-0.877)	0.009	0.725 (0.587-0.895)	0.003

The results showed that the odds of stage IV osteosarcoma were lower for females (OR = 0.654, 0.565-0.757) or those treated in southern region facilities (OR = 0.669, 0.466-0.962). Conversely, the odds of stage IV osteosarcoma were higher for patients on Medicaid (OR = 1.449, 1.030-2.038) or Medicare (OR = 1.662, 1.104-2.500).

The results showed that the odds of stage IV chondrosarcoma were lower for females (OR = 0.823, 0.682-0.992), those with private insurance, (OR = 0.481, 0.317-0.732), those treated at academic/research facilities (OR = 0.483, 0.273-0.854), and patients living 50-99 miles (OR = 0.667, 0.484-0.919) or 100+ miles from their treating facility (OR = 0.601, 0.423-0.855). Conversely, the odds of stage IV chondrosarcoma were higher for older patients (OR = 1.020, 1.009-1.030) and those living in areas with annual incomes between $48,000 and $62,999 (OR = 1.435, 1.008-2.043).

The results showed that the odds of stage IV Ewing sarcoma were lower for those with private insurance (OR = 0.419, 0.212-0.732). Conversely, the odds of stage IV Ewing sarcoma were higher for older patients (OR = 1.016, 1.001-1.030).

For chordomas, besides an increase in odds of stage IV cancer at diagnosis for those with unknown insurance status (OR = 4.376, 1.479-12.943) compared to those with private insurance, there were no factors that showed statistically significant associations between increased or decreased risk of stage IV cancer at diagnosis. 

## Discussion

This study demonstrated that females had a lower risk for stage IV bone cancer at diagnosis overall and for those with osteosarcoma or chondrosarcoma histology. Previous studies have reported that male patients have decreased utilization of healthcare services which may lead to the diagnosis of diseases such as cancer at later stages [11]. Additionally, the presenting symptoms of bone cancer are often nonspecific and may range from intermittent variable intensity localized pain and swelling to a palpable mass or even pathological fractures [12]. In addition to decreased overall use of healthcare, men often ignore or underreport their symptoms which may further delay the diagnosis of primary bone cancer [13].

In this study, when compared to uninsured patients, those with private insurance had lower odds of stage IV bone cancer at diagnosis overall and for those with chondrosarcoma or Ewing sarcoma histology, but those with Medicaid or Medicare had increased odds of stage IV bone cancer at diagnosis overall and for those with osteosarcoma histology. Previous studies have noted that patients without insurance or those utilizing Medicaid as their primary payor were more likely to be diagnosed with late-stage cancers [14]. A study by Plascak et al. in 2014 reported that increases in primary care provider density were associated with a decreased late-stage diagnosis of cancers of the prostate, breast, skin, pharynx, oral cavity, and bronchus/lungs. These changes were most significant in patients with private insurance but were only marginally improved or even worsened in patients with other forms of insurance [14]. Historically, uninsured patients have difficulty accessing and paying for care due to lack of coverage and the increasing costs of medical care in the United States, however, patients on Medicaid also have reduced access to care compared to privately insured patients [15]. In a 2019 meta-analysis, patients with Medicaid had a 1.6x lower likelihood of successfully scheduling a primary care appointment and a 3.3x lower likelihood of successfully scheduling a specialty appointment compared to patients with private insurance [15].

This study also identified that compared to community cancer programs, odds of stage IV bone cancer at diagnosis overall were lower for those treated at comprehensive cancer programs, academic/research facilities, or integrated cancer network programs, lower for chondrosarcoma patients treated at academic/research facilities, and lower for osteosarcoma patients treated in southern region facilities. According to the American College of Surgeons, community cancer programs are relatively small facilities that diagnose less than 500 malignancies per year, and comprehensive community cancer programs are somewhat larger with a minimum of 500 new cancer diagnoses per year [16]. Previous studies have reported that treatment volume may have a significant impact on patient treatments and survival in various types of cancer or other diseases [17-20]. As bone tumors comprise <1% of all cancer diagnoses, it is reasonable to assume that facilities with a higher number of patients presenting with cancer or greater integration of cancer services would be more able to identify bone tumors at early stages [8]. Physicians at these facilities would have greater exposure and experience with these rare cancers and therefore may be more likely to identify and make a diagnosis at an earlier stage. Regarding census region and cancer staging at diagnosis, other studies on cancers have found that odds of advanced-stage cancer at diagnosis are lowest in patients diagnosed and treated at facilities within the southeast region of the United States and increased in those located in mountain or pacific census regions [21].

Additionally, this study identified that those living in urban areas had overall increased odds of stage IV bone cancer at diagnosis. Prior studies have noted that urban areas may have an increased risk of late-stage cancer at diagnosis when compared to metropolitan or moderately rural regions [22]. It is suggested that populations in these areas have high frequencies of socioeconomic and health disparities such as financial difficulties, decreased access to care, decreased frequency of care utilization, decreased cancer awareness, differences in cancer diagnosis, and more time-space constraints when trying to access care [22]. This high frequency of healthcare disparities in highly urbanized areas exemplifies the need for urban-based medical interventions and education programs that specifically target the most vulnerable populations in these areas.

Regarding cancer histology, this study found that patients with chondrosarcomas or chordomas had decreased proportions of stage IV cancer at diagnosis while patients with Ewing sarcoma had increased proportions of stage IV cancer at diagnosis. Prior to filtering of cases with tumors that were not staged I or IV, stage IV tumors were diagnosed in 15.4% of osteosarcoma patients, 6.1% of chondrosarcoma patients, 25.6% of Ewing sarcoma patients, and 2.4% of chordoma patients. Osteosarcoma is the most common form of primary bone cancer and due to variations in grade and its numerous subtypes, it has a significant range with regard to the rate of metastasis at diagnosis [23]. A Surveillance, Epidemiology, and End Results (SEER) study of 7104 patients with osteosarcoma diagnosed between 1999-2008 reported an average rate of distant tumor spread to be 17.4% [24]. Conversely, chordomas are slow-growing tumors that infrequently metastasize to locations such as lymph nodes, lungs, or the liver [25]. A SEER study of 808 patients with chordomas diagnosed between 1973-2014 found a rate of distant tumor spread to be 7.7% [26]. Chondrosarcomas also are often slow-growing tumors but may present with rare subtypes that can grow rapidly and spread to other sites [27]. Another SEER study of 2890 patients with chondrosarcomas diagnosed between 1973-2003 found a rate of distant tumor spread to be 7.39% [28]. Finally, Ewing sarcoma is a rare tumor that primarily affects children and has a peak incidence in patients 15 years of age. Ewing sarcoma histologically is an undifferentiated small round cell sarcoma and has been reported to have metastases at diagnosis in 25-32% of cases [12,29-30]. All of these findings were similar to the results of this study.

Regardless of cancer histology, early detection is associated with better patient outcomes. Detection at early stages is associated with decreased need for deforming or highly invasive surgeries such as limb amputation or large field resections. Additionally, early detection allows for a shorter duration of treatment when chemotherapy or radiation therapy is used. Staging has a significant impact on patient prognosis as patients with early-stage tumors when compared to those with advanced-stage tumors often have significantly decreased mortality at five years after diagnosis. Aside from cancer histology, other characteristics such as the site of the primary tumor and tumor size also play significant roles in determining patient prognosis [31-33].

Those patients who traveled 50+ miles to their treatment facility had decreased odds of stage IV bone cancer at diagnosis overall and for those with chondrosarcoma histology. This contrasts with other studies which reported that greater travel distance to the treatment facility was associated with more advanced-stage cancers [34]. However, other studies have noted that increased travel distance to the treatment facility may be associated with presentation at higher volume centers [34-35]. Therefore the findings of this study may be more a reflection of patient-selective treatment at higher volume centers due to greater treatment options or more favorable socioeconomic factors than a representation of distance and its effects on cancer stage at diagnosis.

Finally, this study identified that increasing age at diagnosis showed marginally increased odds of stage IV cancer at diagnosis for patients with Ewing sarcoma and chondrosarcoma histology but overall, marginally reduced odds of late-stage cancer when all histologies were combined. These results were statistically significant but as their odds ratio was so close to one their clinical significance may be minimal. As noted above, Ewing sarcoma is primarily diagnosed in younger patients, thus increasing age may delay diagnosis [12,29-30]. For chondrosarcomas, increasing age may be associated with an increased risk of genetic mutations that may be associated with a higher risk of developing rare subtypes that tend to metastasize [27]. Overall reduced odds of stage IV at diagnosis in adults may be associated with increased frequency of primary care visits or trends in imaging studies in the US [22]. Between 2000 and 2016 there was a significant increase in the use of imaging studies such as x-rays, CT scans, and MRIs which was most pronounced in adult and elderly patient populations [36]. As these imaging modalities are increasingly utilized and become increasingly sensitive, it may be that incidental diagnosis in these patient populations is increasing and thus decreasing the odds of metastasis at diagnosis.

Limitations

The NCDB is a powerful database that records approximately 70% of all new cancer diagnoses in the United States each year, but it is not a population-based database like SEER. While the NCDB allows for the extraction and analysis of patient, geographic, and socioeconomic data in many hospitals, this data is not recorded if hospitals are not participants in the NCDB registry [37]. This study is also limited by the relatively low sample size and frequencies of different cancer histologies at different stages. While many multivariable relationships were noted in the integrated analysis, these relationships were only partially represented in the individualized histology analyses or may have even been reversed. These discrepancies may in part be due to the increased statistical power of having a larger number of cases in the integrated analysis but may also have been due to differences originating from the highly heterogenous behavior of different primary tumor histologies.

## Conclusions

In conclusion, this study identified that factors such as male sex, use of Medicare or Medicaid, treatment at smaller facilities, and Ewing sarcoma or osteosarcoma histology are associated with increased odds of stage IV bone cancer at diagnosis. Bone tumors overall are a rare form of primary malignancy, but it is still important to recognize the significant effects socioeconomic and geographic factors have on the staging of bone cancer at diagnosis. Earlier recognition is associated with improved prognosis, decreased patient suffering, and lower financial, physical, and social costs in patients with these potentially debilitating, disfiguring, and deadly tumors. If physicians are made aware of these disparities and instructed to have low clinical suspicion for patients with presenting symptoms that are often non-specific and intermittent or persistent, these patients may be identified and diagnosed with minimal disease progression. Despite their rarity, bone cancers are associated with significant cost and suffering, and prompt identification may help reduce these consequences in those who are disproportionately affected.
